# Antimicrobial Effects of Tannic Acid Combined with Plasma-Activated Water and Their Application in Strawberry Preservation

**DOI:** 10.3390/foods14132216

**Published:** 2025-06-24

**Authors:** Zhixiang Hu, Zhenyang Hu, Huan Zhang, Zhilong Yu, Yunfei Xie

**Affiliations:** 1 State Key Laboratory of Food Science and Technology, Jiangnan University, No. 1800 Lihu Avenue, Wuxi 214122, China; 6220111183@stu.jiangnan.edu.cn (Z.H.); 7210112089@stu.jiangnan.edu.cn (Z.H.); 7230112053@stu.jiangnan.edu.cn (H.Z.); zhilong.yu@jiangnan.edu.cn (Z.Y.); 2 School of Food Science and Technology, Jiangnan University, No. 1800 Lihu Avenue, Wuxi 214122, China

**Keywords:** plasma-activated water, natural antibacterial agents, antibacterial mechanism, strawberry preservation

## Abstract

This study investigated the combined antibacterial effects of PAW with natural antimicrobial agents and further examined the impact of this technology on postharvest strawberry preservation. The optimal PAW preparation condition was determined at 50 min at 400 W, although PAW alone showed limited efficacy against *Staphylococcus aureus* and *Escherichia coli*. Among the five selected natural antimicrobial agents, the 1% tannic acid–PAW combined treatment demonstrated optimal bactericidal performance, achieving reductions of 3.62 log CFU/mL for *S. aureus* in 20 min and 5.13 log CFU/mL for *E. coli* in 8 min. The results revealed membrane damage in both *S. aureus* and *E. coli*, with leakage of intracellular proteins and nucleic acids, decreased membrane protein content, and cellular shrinkage and collapse observed morphologically. Increased MDA content indicated membrane lipid peroxidation, while elevated intracellular H_2_O_2_ and ROS levels resulted from oxidative stress induced by PAW’s reactive species. Tannic acid reduced SOD and CAT enzyme activities, impairing bacterial antioxidant capacity, and PAW further exacerbated the decline in SOD and CAT activities, intensifying oxidative stress and disrupting bacterial physiological balance. In strawberry preservation applications, the combined treatment reduced surface microbial loads, decreased mold incidence and weight loss, slowed the deterioration of color, firmness, and edible quality, and enhanced antioxidant capacity. The results suggest that the tannic acid–PAW combined treatment offers a promising strategy for enhancing microbial safety and extending the shelf life of strawberries.

## 1. Introduction

Strawberries are widely favored by consumers for their rich nutritional value and unique flavor. However, they are highly susceptible to postharvest quality deterioration due to microbial infection and physiological metabolism, causing postharvest losses as high as 30–40% annually [1]. In addition, there have been multiple foodborne outbreaks associated with strawberries [2,3]. Although traditional chemical fungicide treatments can effectively control decay and reduce pathogen attachment, they pose issues such as pesticide residues and environmental pollution. Developing safe and green preservation technologies is of great importance for the sustainable development of the strawberry industry.

Plasma-activated water (PAW), as a derivative application of low-temperature plasma technology, transfers reactive species generated by plasma into the liquid phase, combining the dual advantages of plasma’s high sterilization efficiency and the convenience of liquid treatment [4]. As an emerging green sterilization technology, PAW has attracted widespread attention in recent years in fields such as food preservation. Its unique bactericidal properties stem from the synergistic effects of complex reactive components. Studies have shown that PAW primarily contains hydrogen peroxide, nitrites, ozone, and short-lived reactive species such as hydroxyl radicals and superoxide anions [5]. These substances exert antibacterial effects through multiple mechanisms, including oxidative damage to cell membranes, disruption of protein structures, and interference with microbial metabolism [6]. Due to the generation of the aforementioned active species, the physical properties of PAW will also change, such as a significant increase in conductivity. So far, PAW has shown significant inhibitory effects against various foodborne pathogens [7]. Studies indicate that PAW can inactivate up to 5-log microbial populations on lettuce surfaces without negatively affecting its sensory qualities (e.g., color and texture) [5]. For strawberry preservation, PAW treatment reduced *S. aureus* by 1.6–2.3 log and 1.7–3.4 log on days 0 and 4 of storage, respectively [8]. Compared to traditional chlorine disinfection (100–200 ppm sodium hypochlorite), PAW not only achieves comparable sterilization efficacy but also avoids chlorine residues and off-flavor issues [9], while preventing significant changes in sensory characteristics, including appearance, color, flavor, texture, and overall acceptability [10]. However, PAW technology still faces several challenges in practical applications: (1) the short half-life of active components limits storage stability [11]; (2) insufficient penetration into complex food matrices; (3) energy consumption and cost issues in large-scale production; (4) insufficient molecular-level evidence for its mechanisms. To address these issues, many studies focus on the synergistic application of PAW with other technologies (e.g., ultrasound [12] and natural antimicrobials [13]), novel reactor designs, and the development of stabilization techniques for active components.

Natural antibacterial agents (such as essential oils, polyphenolic compounds, and antimicrobial peptides) have gained considerable attention due to their wide availability, biodegradability, low toxicity, and excellent biocompatibility. Research indicates that many natural active components (e.g., tea polyphenols, chitosan, and thymol) can inhibit microbial growth by disrupting cell membranes, inhibiting enzyme activity, or interfering with DNA replication [14]. However, natural antibacterial agents still face certain limitations in practical applications, such as poor water solubility, narrow antimicrobial spectra, or reduced activity in complex food systems, which restrict their effectiveness when used alone. For example, many natural antibacterial agents have been demonstrated for their use as fruit sanitizers. ε-Polylysine hydrochloride (ε-PL) is a homopolyamide produced by *Streptomycetaceae* and *ergot* fungi. It forms amide bonds (peptide bonds) with adjacent monomer α-carboxyl groups (-COOH) through the ε-amino group (-NH_2_) of lysine, forming a straight chain polymer of 25–35 lysine residues. ε-PL exhibits broad-spectrum antimicrobial activity against Gram-negative and Gram-positive bacteria, yeasts, and molds [15]. Citric acid is a weak organic acid naturally found in limes, oranges, and lemons that can act as an antimicrobial agent by delaying the growth of bacteria, molds, yeasts, and fungi [16]. Lysozyme is an antimicrobial peptide that can act on cell membranes to disrupt bacteria [17], while phytic acid is the primary storage form of phosphorus in plant seeds and grains, exhibiting bactericidal effects against planktonic *E. faecalis* [18]. Tannic acid (TA), the most important representative of hydrolyzable tannins, consists of a central monosaccharide core (commonly glucose) and exhibits inhibitory activity against viruses and bacteria as a weak acid [19].

The aims of this work were to investigate the synergistic antibacterial effects between PAW and natural antimicrobial agents. Five selected natural antimicrobials were combined with PAW under different treatment conditions to evaluate their combined antimicrobial efficacy. Given that different natural antimicrobials possess distinct antibacterial mechanisms, their interactions with PAW may result in either synergistic or antagonistic effects, inherently leading to varying degrees of synergistic efficacy. Therefore, potential synergistic mechanisms between PAW and natural antimicrobial agents were examined. Finally, the effects of combined PAW–tannic acid washing treatment on strawberry preservation were explored, to confirm its potential in improving fruit safety and extending shelf life.

## 2. Materials and Methods

### 2.1. Materials

*S. aureus* (ATCC6538) was purchased from Beijing Beina Technology Co., Ltd. (Beijing, China), *E. coli* (CICC24187) was purchased from Shanghai Luwei Technology Co., Ltd. (Shanghai, China), and Luria–Bertani (LB) broth, Luria–Bertani (LB) agar, and potato dextrose agar (PDA) were purchased from Qingdao Hope Bio-Technology Co., Ltd. (Qingdao, China). ε-PL, citric acid, lysozyme, phytic acid, and tannic acid were purchased from Sinopharm Chemical Reagent Co., Ltd. (Shanghai, China). 2′,7′-Dichlorodihydrofluorescein diacetate (DCFH-DA) fluorescent probe was purchased from Sigma Aldrich (St. Louis, MO, USA); 2.5% glutaraldehyde solution was purchased from Shanghai Yuanye Bio-Technology Co., Ltd. (Shanghai, China).

### 2.2. Bacteria Cultivation

*S. aureus* and *E. coli* were inoculated into LB broth medium, activated at 37 °C for 24 h, and then subcultured in fresh medium to the exponential phase. The bacteria were collected by centrifugation (2000 rpm, 25 °C, 5 min), washed, and resuspended in sterile phosphate-buffered saline (PBS) to adjust the concentration to 10^9^ CFU/mL for subsequent experiments.

### 2.3. PAW Inactivation and Synergistic Treatment

PAW was produced by treating deionized water (10 L) using a plasma-activated water generator (SUMAN Plasma Technology Co., Ltd., Nanjing, China) at 400 W. Different PAW samples were prepared by varying the power (100, 200, 300, and 400 W) and treatment time (10, 20, 30, 40, and 50 min). Bacterial suspensions were diluted to 10^6^ CFU/mL using different PAW samples. For antimicrobial agent treatments, after testing at different concentrations, bacterial suspensions were separately treated with different natural antimicrobial agents (1%, *w*/*v*), including ε-PL, citric acid, lysozyme, phytic acid, and tannic acid, and diluted to 10^6^ CFU/mL. For combination treatment, natural antimicrobial agents were dissolved in PAW. Each mixture was incubated for different time intervals (2, 4, 6, 8, 10, 15, and 20 min). The treated bacterial suspensions were serially diluted 10-fold. A 100 μL aliquot of each dilution was spread on LB agar plates and incubated at 37 °C for 24 h before colony counting [20].

### 2.4. Determination of Nucleic Acid and Protein Leakage

Based on a reported method [21], the activated *S. aureus* and *E. coli* suspensions (10^9^ CFU/mL) were treated with PAW, TA, and 1% tannic acid–PAW combination, and the samples were centrifuged at 8000× *g* (Sorvall ST16R, Thermo Fisher Scientific Inc., Waltham, MA, USA) for 10 min. The supernatant was collected and measured for nucleic acid and protein content using a UV spectrophotometer (UV-1800, Shimadzu Corporation, Kyoto, Japan) at 260 nm and 280 nm. Sterile PBS solution was used as a blank reference.

### 2.5. Total Cellular Protein Concentration

The bacterial suspensions after different treatments were centrifuged at 8000× *g* for 10 min. After supernatant removal, the pellets were resuspended in sterile PBS. Cell disruption was performed using an ultrasonic homogenizer (SN-P150, Shenna Instruments, Shanwei, China). Following cell lysis, total cellular protein concentration was determined using a BCA protein assay kit (Shanghai Yuanye Bio-Technology Co., Ltd., Shanghai, China), with absorbance measured at 562 nm.

### 2.6. Morphological Observation

Bacterial precipitates were fixed with 2.5% glutaraldehyde at 4 °C for 12 h and washed three times with sterile PBS. Samples were then dehydrated through a graded ethanol series (30%, 50%, 70%, 80%, 90%, 95%, and 100%, *v*/*v*) with 10 min per step, air-dried for 24 h, sputter-coated with gold, and examined by using a multifunctional field-emission scanning electron microscope (SEM, Ultim Max65, Oxford Instruments, Oxford, UK). Untreated cells were used as controls.

### 2.7. Determination of Malondialdehyde (MDA) Content

The MDA content was determined according to a reported method [22]. A 5 mL aliquot of treated bacterial suspension was mixed with 3 mL of 10% trichloroacetic acid solution, followed by centrifugation at 8000× *g* for 5 min. Then, 1 mL of 0.67% thiobarbituric acid was added to 0.8 mL of the supernatant, thoroughly mixed, and heated in a boiling water bath for 15 min before immediate cooling in an ice bath to terminate the reaction. Finally, the absorbance was measured at 450, 532, and 600 nm.

### 2.8. Reactive Oxygen Species (ROS) and Antioxidant Enzyme Activity Measurements

Hydrogen peroxide (H_2_O_2_) content was measured colorimetrically using a hydrogen peroxide assay kit (Beijing Solarbio Science & Technology Co., Ltd., Beijing, China). ROS content was determined by staining with 10 μM DCFH-DA for 4 h. Intracellular ROS levels were detected at 525 nm using a fluorescence spectrophotometer (UV-Vis, Shimadzu Corporation, Kyoto, Japan). Antioxidant enzyme activities were measured using superoxide dismutase (SOD) and catalase (CAT) assay kits (Shanghai Yuanye Bio-Technology Co., Ltd., Shanghai, China).

### 2.9. Application in Strawberry Preservation

Fresh strawberries of uniform size and color were purchased from a local market in Wuxi, China. The strawberries were evenly divided into three groups: a water-treated group, sodium hypochlorite-treated group, and tannic acid–PAW combined treatment group. The three groups of strawberries were immersed in sterile water, 50 ppm sodium hypochlorite solution, and the combined treatment solution for 20 min, respectively. After treatment, the strawberry surfaces were blotted dry with sterile gauze and air-dried in a laminar flow hood for 20 min. The samples were then stored in PET containers (20 cm × 10 cm × 8 cm) at 25 °C for storage.

For each treatment group, strawberries were randomly selected and placed into sterile bags containing sterile water, followed by homogenization for 2 min. The resulting homogenate was filtered through sterile gauze, and 1 mL of the filtrate was collected. Serial dilutions were prepared, and appropriate aliquots were plated onto LB agar and potato dextrose agar (PDA) for microbial enumeration. LB plates were incubated at 37 °C for 24 h to determine total bacterial counts, while PDA plates were incubated at 28 °C for 3–5 d for mold and yeast enumeration.

The weight and mold count of strawberries were recorded at each storage time point and compared with the initial values to calculate the strawberry weight loss percentage and mold incidence percentage. Three fruits were taken from each group, and a colorimeter (Hunter Assoc. Laboratory Inc., Reston, VA, USA) was used to take the color parameters of the samples. The hardness was measured using a texture analyzer (Stable micro system TA.XT plus Texture, London, UK).

The titratable acidity, total soluble solids (TSS), and total phenolic content of strawberries were determined based on the reported method with some modifications [23]. For each treatment group, strawberries were randomly selected and homogenized with 30 mL sterile water for 2 min, followed by centrifugation at 7000 rpm for 10 min at 4 °C to obtain the supernatant. Titratable acidity was determined by acid–base neutralization titration using 0.1 M NaOH, with the titration endpoint set at pH 8.1, and expressed as a mass fraction of tartaric acid. TSS content was analyzed using an Abbe refractometer (WAY-2S, Shanghai ShenGuang Instrument Co., Ltd., Shanghai, China) and expressed as a percentage (%). For total phenolic content determination, 1 mL of the supernatant was mixed with 0.5 mL Folin–Ciocalteu reagent and 4 mL of 7.5% Na_2_CO_3_ solution and then diluted with distilled water to a final volume of 10 mL. After thorough mixing, the solution was incubated at 37 °C in a water bath for 30 min in the dark. The absorbance at 760 nm was measured.

The DPPH radical scavenging activity was determined with slight modifications based on the reported method [24]. Three strawberries were randomly selected from each group and homogenized with 30 mL of sterile water for 2 min. Then, 1 mL was taken and homogenized with 1 mL of ethanol solution (80%, *v*/*v*) and centrifuged for 10 min at 25 °C. Then, 1 mL of the supernatant was taken and fully mixed with 1 mL of DPPH ethanol solution (0.1 M) and reacted at room temperature in the dark for 30 min, and the absorbance was measured at 515 nm.

### 2.10. Statistical Analysis

All determinations were repeated at least 3 times, and the experimental results were expressed as means ± standard deviations. Data were analyzed using one-way analysis of variance (ANOVA), and the difference was significant at *p* < 0.05.

## 3. Results and Discussion

### 3.1. Antibacterial Effects and Kinetics of PAW and Different Natural Antimicrobial Agents

**Figure 1:**
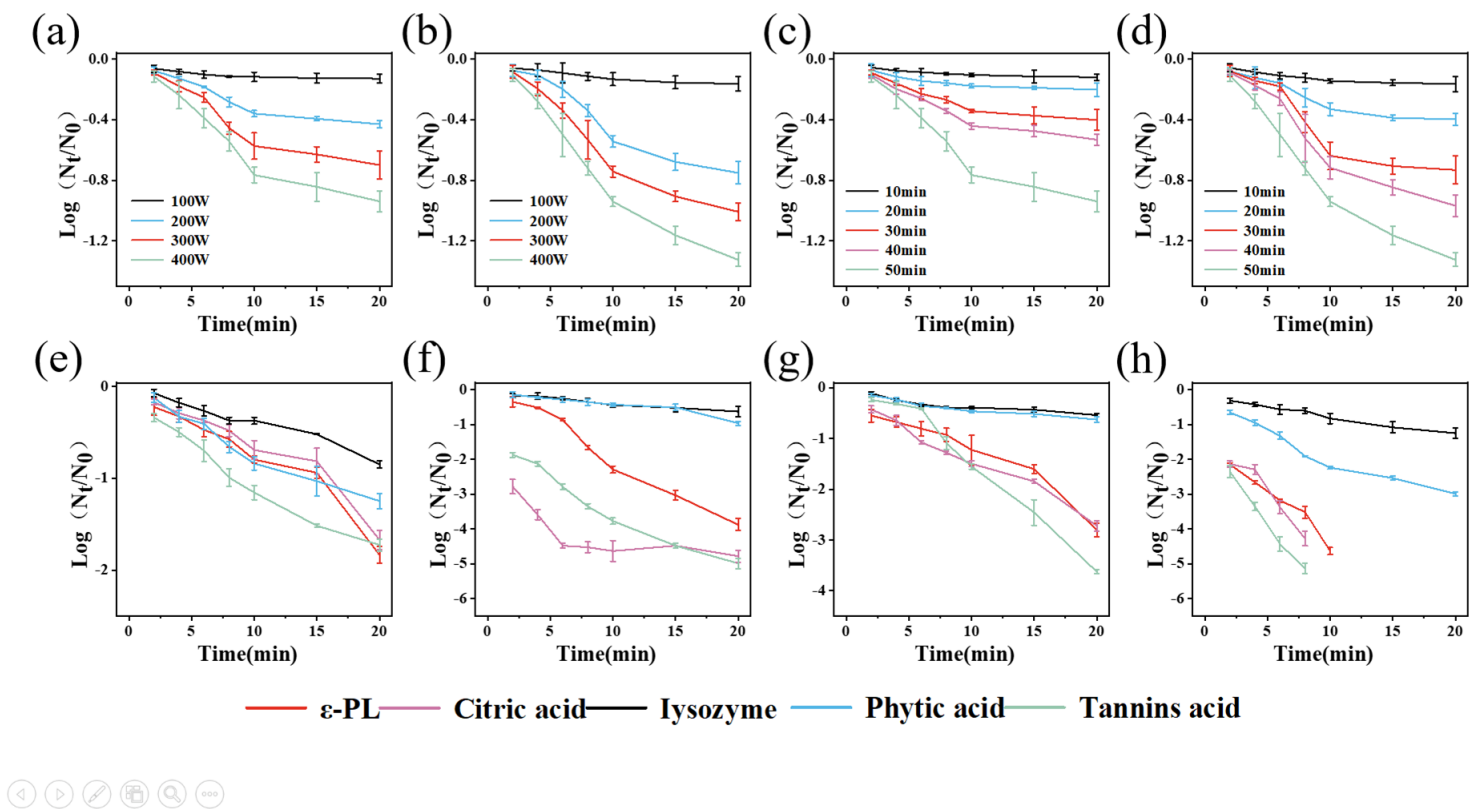
Microbial reduction by PAW produced at different preparation powers ((**a**), *S. aureus*; (**b**), *E. coli*), by PAW produced for different preparation times ((**c**), *S. aureus*; (**d**), *E. coli*), by different natural antimicrobial agents alone ((**e**), *S. aureus*; (**f**), *E. coli*), and by combination treatments of different natural antimicrobial agents with PAW ((**g**), *S. aureus*; (**h**), *E. coli*).

With increased preparation power and time, the bactericidal effect of PAW increased (Figure 1a–d). When the preparation power reached 400 W and the preparation time reached 50 min, the reductions in *S. aureus* and *E. coli* were 0.93 lg CFU/mL and 1.32 lg CFU/mL, respectively. The produced PAW achieved the highest inactivation efficiency and was used for subsequent experiments. Notably, the PAW alone could not meet the practical application requirements in the food industry. Therefore, PAW was combined with different natural antimicrobial agents to enhance its bactericidal effect. The pH of PAW was 3.68, which may affect the solubility of certain antimicrobial agents. Among all the combination treatment groups (Figure 1g,h), the tannic acid–PAW combination exhibited the highest enhancement in antibacterial effect compared to individual natural antimicrobial agent treatments (Figure 1e,f). After combining with PAW, the antimicrobial effects of ε-PL, citric acid, and tannic acid were enhanced, while the improvements for lysozyme and phytic acid were not significant. The 1% tannic acid–PAW combination exhibited the highest antibacterial effect, reducing *S. aureus* by 3.62 lg CFU/mL and *E. coli* by 5.13 lg CFU/mL within 8 min. The results demonstrated that 1% tannic acid could significantly enhance the antibacterial effect of PAW against *S. aureus* and *E. coli*. Therefore, the 1% tannic acid–PAW combination was selected for subsequent studies.

### 3.2. Changes in Bacterial Cell Membrane Permeability and Integrity

**Figure 2:**
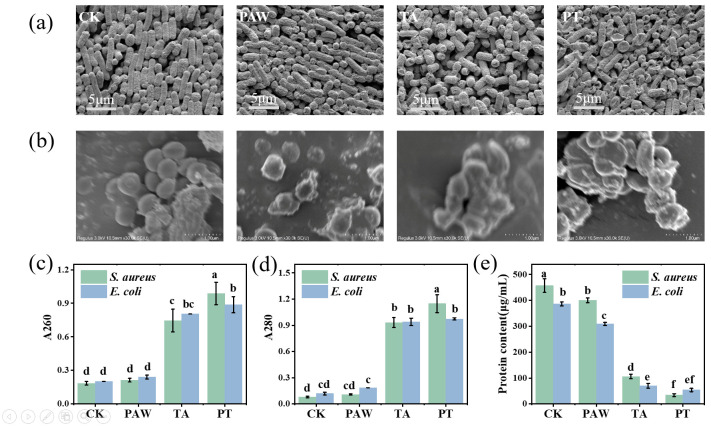
SEM images of *E. coli* (**a**) and *S. aureus* (**b**); changes in nucleic acid (**c**) and protein (**d**) leakage in *S. aureus* and *E. coli*; intracellular protein content in *S. aureus* and *E. coli* cells (**e**). Results with different lower-case letters are significantly different (*p* < 0.05). CK, TA, PAW, and PT represent the control, tannic acid, plasma-activated water, and combined tannic acid–plasma-activated water treatments, respectively.

As shown in Figure 2a,b, SEM observations demonstrated that control *E. coli* and *S. aureus* cells maintained their typical rod-shaped and spherical morphology with a uniform size and intact structure, whereas PAW-treated samples showed increased surface roughness, and TA-treated cells exhibited shrinkage and collapse at rod termini, consistent with previous reports of bacterial morphological changes induced by these treatments [25,26]. The combined treatment of PAW and tannic acid was designated as PT. Notably, PT-treated cells displayed more severe damage characterized by extensive surface irregularities, size heterogeneity, and generalized cellular collapse beyond polar regions, indicating that the combined treatment synergistically enhanced bacterial destruction through comprehensive structural compromise.

To investigate the effects of PAW combined with tannic acid on cell membrane permeability, the leakage of biomacromolecules (e.g., nucleic acids and proteins) from *S. aureus* and *E. coli* was measured as an indicator of membrane damage. As shown in Figure 2c, no significant difference (*p* > 0.05) was observed in absorbance at 260 nm between the PAW treatment alone and the control group, indicating that PAW treatment did not induce remarkable nucleic acid leakage in either *S. aureus* or *E. coli*, which is consistent with previous findings [21]. In contrast, both the tannic acid treatment and the combined treatment resulted in significantly higher (*p* < 0.05) nucleic acid leakage compared to the PAW treatment alone. As shown in Figure 2d, the same trend was observed for absorbance at 280 nm, where the tannic acid treatment and combined treatment resulted in significant protein leakage in *S. aureus* and *E. coli*. Notably, for *S. aureus*, the leakage amount with the combined treatment was significantly higher than with the tannic acid treatment alone (*p* < 0.05), while for *E. coli*, there was no significant difference in the leakage amount between the combined treatment and tannic acid treatment alone (*p* > 0.05). This may be because tannic acid itself has a destructive effect on bacterial cell walls, directly binding to the peptidoglycan layer and disrupting cell wall integrity [27]; PAW has a similar effect, capable of damaging the bacterial cell wall and membrane structure [28]. The combined treatment further enhanced the damaging effect of tannic acid on the cell membrane. Since *E. coli* has a thinner cell wall and the effect is more focused on the cell membrane, the combined treatment did not increase membrane damage compared to the tannic acid treatment alone.

Normal cellular physiological activities rely on various functions of proteins, so intracellular protein concentration can reflect whether cellular physiological functions are at normal levels. As shown in Figure 2e, PAW, tannic acid, and combined treatments all caused significant decreases in cellular protein concentration (*p* < 0.05). Tannic acid can damage proteins on bacterial cell membranes, including inactivating and destroying ATPases located in the membrane. When these processes are disrupted, bacteria cannot obtain the energy required for cell growth and function [26]. In addition, tannic acid can reduce the expression levels of several proteins involved in peptidoglycan synthesis, leading to shrinkage of the outermost cell wall and disruption of membrane integrity [29,30]. Thus, it is possible that the combined treatment can interact with multiple sites in bacterial cell membranes, thereby inhibiting the activity of various enzymes on the membranes of *S. aureus* and *E. coli*, impairing protein synthesis and compromising membrane integrity.

### 3.3. Impact on ROS and Antioxidant Enzyme Activities in Bacterial Cells

**Figure 3:**
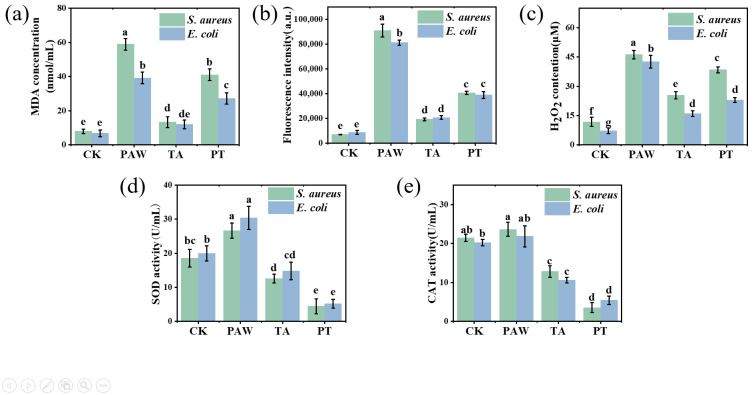
Effects of different treatment durations on MDA content (**a**), ROS content (**b**), H_2_O_2_ content (**c**), SOD enzyme activity (**d**), and CAT enzyme activity (**e**) in *S. aureus* and *E. coli* cells. Results with different lower-case letters are significantly different (*p* < 0.05). CK, TA, PAW, and PT represent the control, tannic acid, plasma-activated water, and combined tannic acid–plasma-activated water treatments, respectively.

The lipid peroxidation levels of *S. aureus* and *E. coli* cell membranes were evaluated by measuring the MDA content. As shown in Figure 3a, the MDA levels in the PAW-treated and synergistically treated groups were significantly higher than those in the blank control and tannic acid-only groups (*p* < 0.05). After treatment with PAW, tannic acid, and the combined treatment, the MDA contents in *S. aureus* were 58.88, 13.28, and 41.06 nmol/mL, respectively. Compared with the control group, the MDA contents in PAW and combined treatments increased by 6.46- and 5.20-fold, respectively. For *E. coli*, the MDA contents after PAW, tannic acid, and combined treatments were 39.22, 11.97, and 27.15 nmol/mL, respectively, representing 4.85- and 3.05-fold increases over the control group for PAW and combined treatments. The tannic acid treatment alone resulted in low MDA content and did not induce lipid peroxidation in *S. aureus* and *E. coli* cell membranes. PAW contains various reactive species, which can oxidize cell membranes and induce lipid peroxidation in both *S. aureus* and *E. coli* [31,32]. The elevated MDA content observed after PAW and combined treatments indicates the occurrence of lipid peroxidation in bacterial membranes.

Measuring intracellular ROS levels can indicate cellular physiological functionality and characterize cellular damage [31]. ROS can induce the formation of lipid radicals from unsaturated fatty acids in cell membranes through free radical reactions. These subsequently undergo chain reactions to form lipid hydroperoxides, which ultimately decompose to produce malondialdehyde (MDA). The fluorescent probe DCFH-DA was used to detect intracellular ROS. As shown in Figure 3b,c, the control group exhibited significantly lower ROS and H_2_O_2_ levels compared to the treated groups (*p* < 0.05). Among treated groups, PAW-treated cells displayed the highest fluorescence intensity and H_2_O_2_ levels, due to reactive components in PAW that induce lipid peroxidation of bacterial membranes while generating substantial intracellular ROS through oxidative reactions [32]. Additionally, H_2_O_2_ in PAW can penetrate cell membranes. The combined treatment group showed significantly lower ROS and H_2_O_2_ levels than PAW alone (*p* < 0.05), likely due to tannic acid’s inherent antioxidant properties that partially reduce reactive components in PAW [33], diminishing its oxidative capacity. However, the combined treatment still maintained significantly higher ROS and H_2_O_2_ levels compared with controls (*p* < 0.05), indicating residual reactive components that induced membrane lipid peroxidation via oxidative stress in *S. aureus* and *E. coli* cells. Furthermore, the dual disruptive action on cell walls and membranes caused by tannic acid rapidly creates cellular entry channels, enhancing penetration of reactive components and increasing intracellular ROS levels.

As shown in Figure 3d, the SOD activity of the control for *S. aureus* was 18.52 U/mL. After PAW treatment, the SOD activity increased significantly by 44% (*p* < 0.05). This indicates activation of the bacterial defense system that elevated both SOD and CAT activity levels, which is consistent with previous reports [34]. In contrast, tannic acid alone and the combined treatment significantly decreased SOD activity of *S. aureus* by 32% and 76%, respectively (*p* < 0.05). PAW-treated *E. coli* showed an SOD activity of 30.37 U/mL, representing a significant 52% increase compared to the control (*p* < 0.05). Tannic acid alone and the combined treatment resulted in *E. coli* SOD activities of 14.81 and 5.19 U/mL, showing significant decreases of 25.95% and 74.05%, respectively, compared to the control (*p* < 0.05). Similar results were observed for CAT activity (Figure 3e). The decreased SOD and CAT activities under tannic acid treatment may relate to their catalytic mechanisms. SOD catalysis occurs through alternating electron transfer of metal ions, with iron being one of SOD’s three metal cofactors; iron porphyrin serves as CAT’s cofactor and is equally crucial for its catalysis, making iron essential for optimal bacterial growth. As a polyphenol, tannic acid’s catechol groups can chelate ferric iron from the environment [27], potentially reducing SOD and CAT activities in *S. aureus* and *E. coli* while also affecting other iron-dependent physiological requirements. During combined treatment, the significant SOD activity decrease may result from tannic acid’s membrane-disrupting effects increasing permeability, coupled with its impact on SOD and CAT enzymes.

### 3.4. Effect of Tannic Acid–PAW Combined Treatment on Strawberry Preservation

**Figure 4:**
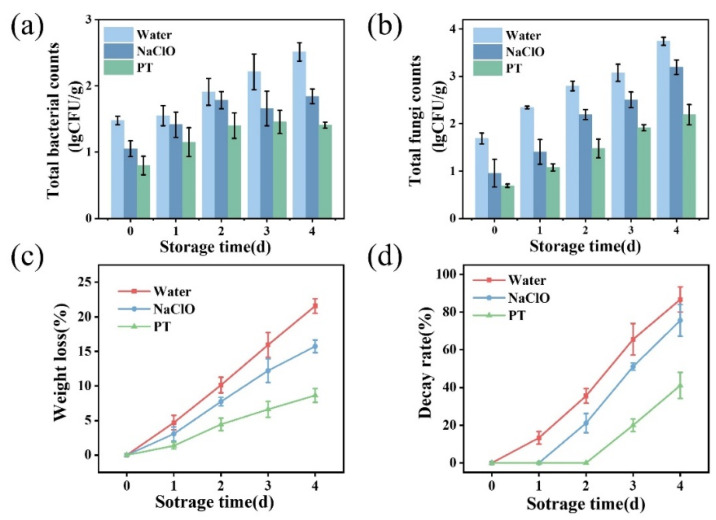
Changes in bacterial (**a**) and fungal (**b**) counts on strawberries following different treatments and effects of different treatments on strawberry weight loss rate (**c**) and decay rate (**d**). PT indicates the combined treatment of 1% tannic acid and plasma-activated water.

NaClO is a traditional disinfectant widely used in tap water as well as in the disinfection of fresh fruits and vegetables, with its concentration typically regulated at 50–200 ppm. However, due to concerns about chlorine residues, researchers have been actively seeking alternatives to chlorine-based disinfectants. Therefore, this study selected NaClO as the control treatment. As shown in Figure 4a, the bacterial count on strawberries was approximately 1.48 lg CFU/g on day 0. After combined treatment, the bacterial count decreased below the detection limit, while the sodium hypochlorite treatment group showed only a slight reduction. During storage, the water-washed control group exhibited exponential bacterial growth, indicating no inhibitory effect. The sodium hypochlorite group demonstrated good antibacterial effects initially (0–2 days), but bacterial growth continued during later stages (3–4 days). For the combined treatment group, bacterial counts remained below detection limits on days 0 and 1 and were significantly lower than the water-washed group on day 4 (*p* < 0.05). Strawberries are commonly susceptible to fungal and yeast infections caused by *Botrytis cinerea*, *Alternaria* spp., and *Candida* spp. Regarding fungal inhibition (Figure 4b), the initial fungal load was 1.69 lg CFU/g on day 0, increasing to 3.60 lg CFU/g by day 4 in the control. Although sodium hypochlorite reduced fungal counts, they still reached 3.04 lg CFU/g by day 4. The combined treatment achieved 0.75 lg CFU/g initially and 2.07 lg CFU/g on day 4, significantly lower than both the control and sodium hypochlorite groups (*p* < 0.05), demonstrating superior antifungal efficacy. The combined treatment showed optimal antimicrobial effects, significantly outperforming both sodium hypochlorite and water-washing in slowing fungal growth, indicating synergistic inhibition of bacterial and fungal growth through tannic acid’s antimicrobial properties and PAW’s oxidative effects. While sodium hypochlorite exhibited good short-term sterilization, its long-term antimicrobial capacity was limited, particularly against fungi.

As shown in Figure 4c, weight loss increased over time, with the slowest loss rate observed in the combined treatment group, followed by the sodium hypochlorite and water-washed groups. The results suggest the combined treatment not only reduced surface microbes but also prevented weight loss. Figure 4d shows mold incidence increased during storage, remaining lowest in the combined treatment (41% on day 4), representing 53% and 46% reductions compared to the water-washed and sodium hypochlorite groups, respectively. This demonstrates significant inhibition of spoilage microorganisms by the combined treatment.

**Figure 5:**
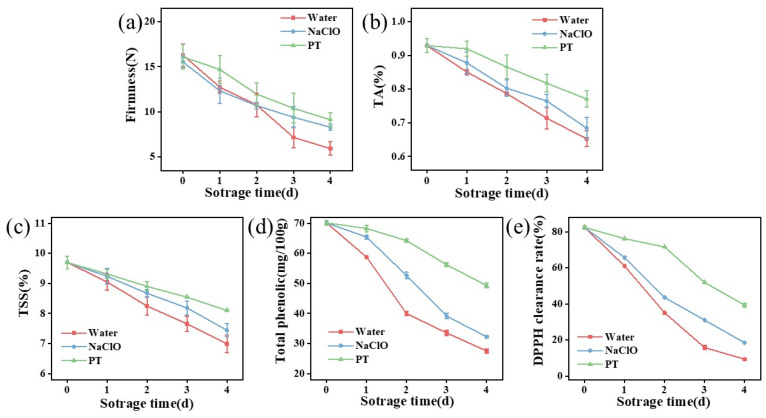
Effects of different treatments on firmness (**a**), titratable acidity content (**b**), total soluble solid content (**c**), total phenolic content (**d**), and DPPH scavenging rate (**e**) of strawberry samples. TA indicates titratable acidity, while TSS means total soluble solids. PT indicates the combined treatment of 1% tannic acid and plasma-activated water.

As shown in Table 1, the combined treatment group exhibited minimal color changes and optimal stability. Comparatively, the synergistic group showed relatively higher L* and a* values. This is likely due to inhibited polyphenol oxidase activity in strawberries [35,36], delaying color changes in the strawberries. In addition, strawberry firmness decreased during storage (Figure 5a), with the combined treatment maintaining the highest values (9.13 N on day 4), which were 10% and 54% higher than the sodium hypochlorite and control groups (*p* < 0.05).

The combined treatment group initially exhibited higher titratable acidity levels with slower decline (Figure 5b), potentially related to ROS. Under external stress, the expression of enzyme genes affecting organic acid biosynthesis pathways is upregulated [37]. Thus, stimulation by either tannic acid or PAW in the combined treatment enhanced organic acid biosynthesis, converting glucose into ascorbic acid within strawberries. As storage duration increased, strawberry aging led to reduced polysaccharides and organic acids, causing rapid decreases in titratable acidity. Moreover, a higher total soluble solid content was observed in synergistically treated strawberries (Figure 5c), which is likely caused by the modulation of respiratory metabolism during fruit storage [38].

The total phenolic content in all groups decreased with prolonged storage (Figure 5d), indicating oxidation or degradation of phenolic compounds in strawberries. The combined treatment group maintained the highest phenolic content, demonstrating its superior protective effect on these compounds. While PAW treatment alone did not alter total phenolic content [39], tannic acid’s antioxidant properties effectively preserved phenolic substances from oxidation. Notably, the combined treatment group exhibited the highest DPPH radical scavenging activity (Figure 5e), further confirming the optimal retention of antioxidants in strawberries. These results mean that the combined treatment protected the antioxidants in strawberries from oxidation [24].

## 4. Conclusions

In this work, the combined use of 1% tannic acid and PAW achieved the best inhibitory effect against *S. aureus* and *E. coli*. *S. aureus* was reduced by 3.62 lg CFU/mL in 20 min, and *E. coli* was reduced by 5.13 lg CFU/mL in 8 min. With the combined treatment, the cell membranes of *S. aureus* and *E. coli* were damaged, the protein content on the membranes decreased, the permeability of the cell membranes increased, and intracellular proteins and nucleic acids leaked. The cell morphology showed shrinkage and collapse, the MDA content increased, and lipid peroxidation occurred on the cell membranes. The combined treatment increased the intracellular H_2_O_2_ and ROS content, mainly due to the oxidative substances in PAW, while the addition of tannic acid further enhanced their destructive power. After washing strawberries with the combined treatment, the bacterial and fungal number on the surface of the strawberries was reduced. The weight loss, color change, and hardness decline of strawberries were slowed down, and the decline of titratable acid, soluble solid content, total phenol content, and the DPPH scavenging rate of strawberries was reduced. This study presents an effective strategy for strawberry sanitization to extend shelf life. Future research will explore the application of this combined treatment to other fresh produce and further clarify its underlying mechanisms of action.

## Figures and Tables

**Table 1:** Effect of different treatments on the color change in strawberries.

Index		Storage Time (days)				
		0	1	2	3	4
	Water	35.10 ± 1.44 ^aA^	33.30 ± 1.33 ^aA^	31.97 ± 0.75 ^abA^	32.45 ± 2.54 ^abA^	29.98 ± 1.73 ^bA^
L*	NaClO	35.20 ± 0.68 ^aA^	33.54 ± 0.54 ^bA^	30.35 ± 1.00 ^cA^	27.93 ± 0.62 ^dB^	29.68 ± 0.79 ^cA^
	PT	34.25 ± 1.31 ^aA^	34.09 ± 0.50 ^aA^	33.24 ± 4.01 ^aA^	31.62 ± 0.23 ^aA^	32.41 ± 1.87 ^aA^
	Water	38.29 ± 0.94 ^aA^	35.59 ± 3.25 ^aB^	32.46 ± 6.07 ^aA^	24.76 ± 5.02 ^bA^	22.29 ± 2.02 ^bB^
a*	NaClO	37.90 ± 2.10 ^aA^	35.16 ± 1.54 ^abA^	33.51 ± 2.12 ^bA^	27.69 ± 3.70 ^cA^	24.26 ± 0.76 ^cB^
	PT	38.14 ± 0.69 ^abA^	41.3 ± 0.70 ^Ba^	36.07 ± 3.66 ^bA^	30.51 ± 0.77 ^bA^	29.32 ± 2.47 ^cA^
	Water	22.06 ± 0.64 ^aA^	18.05 ± 2.79 ^abA^	16.18 ± 4.47 ^bcA^	16.33 ± 3.46 ^bcA^	11.57 ± 0.53 ^cA^
b*	NaClO	21.52 ± 0.68 ^aA^	19.21 ± 2.94 ^aA^	17.92 ± 3.27 ^abA^	12.47 ± 3.82 ^bA^	13.53 ± 1.66 ^bA^
	PT	21.20 ± 0.35 ^aA^	19.36 ± 3.13 ^abA^	17.81 ± 4.63 ^abA^	15.27 ± 1.48 ^bA^	14.91 ± 2.69 ^bA^

Note: Different labels (a–c) in one row indicate significant differences between different sampling days (*p* < 0.05). Different superscripts (A,B) within a column indicate significant differences among samples (*p* < 0.05). PT indicates the combined treatment of 1% (*w*/*v*) tannic acid and plasma-activated water.

## Data Availability

The original contributions presented in the study are included in the article, further inquiries can be directed to the corresponding author.

## References

[1] Priyadarshi R., Jayakumar A., de Souza C.K., Rhim J.W., Kim J.T. (2024). Advances in strawberry postharvest preservation and packaging: A comprehensive review. Compr. Rev. Food Sci. Food Saf..

[2] Bhullar M., Perry B., Monge A., Nabwiire L., Shaw A. (2021). Escherichia coli survival on strawberries and unpacked romaine lettuce washed using contaminated water. Foods.

[3] Aliakbarlu J., Manafi L., Mortazavi N., Lin L., Kaboudari A. (2025). The antibacterial activity of endolysins against food-borne pathogenic bacteria in vitro and foods. Crit. Rev. Food Sci. Nutr..

[4] Shanker M.A., Khanashyam A.C., Pandiselvam R., Joshi T.J., Thomas P.E., Zhang Y., Rustagi S., Bharti S., Thirumdas R., Kumar M. (2023). Implications of cold plasma and plasma activated water on food texture-a review. Food Control.

[5] Xiang Q., Fan L., Li Y., Dong S., Li K., Bai Y. (2022). A review on recent advances in plasma-activated water for food safety: Current applications and future trends. Crit. Rev. Food Sci. Nutr..

[6] Han Q.-Y., Wen X., Gao J.-Y., Zhong C.-S., Ni Y.-Y. (2023). Application of plasma-activated water in the food industry: A review of recent research developments. Food Chem..

[7] Hou C.-Y., Lai Y.-C., Hsiao C.-P., Chen S.-Y., Liu C.-T., Wu J.-S., Lin C.-M. (2021). Antibacterial activity and the physicochemical characteristics of plasma activated water on tomato surfaces. LWT-Food Sci. Technol..

[8] Ma R., Wang G., Tian Y., Wang K., Zhang J., Fang J. (2015). Non-thermal plasma-activated water inactivation of food-borne pathogen on fresh produce. J. Hazard. Mater..

[9] Los A., Ziuzina D., Boehm D., Cullen P.J., Bourke P. (2020). Inactivation efficacies and mechanisms of gas plasma and plasma-activated water against Aspergillus flavus spores and biofilms: A comparative study. Appl. Environ. Microbiol..

[10] Xiang Q., Liu X., Liu S., Ma Y., Xu C., Bai Y. (2019). Effect of plasma-activated water on microbial quality and physicochemical characteristics of mung bean sprouts. Innov. Food Sci. Emerg. Technol..

[11] Wang Q., Salvi D. (2021). Evaluation of plasma-activated water (PAW) as a novel disinfectant: Effectiveness on Escherichia coli and Listeria innocua, physicochemical properties, and storage stability. LWT-Food Sci. Technol..

[12] Tang W., Sun R., Jiang N., Om A.-S. (2025). Effects of ultrasonication coupled with plasma-activated water cleaning on the sterilization and preservation of fresh crucian carp fillets. LWT-Food Sci. Technol..

[13] Zeraat Pisheh F., Falah F., Sanaei F., Vasiee A., Zanganeh H., Tabatabaee Yazdi F., Ibrahim S.A. (2023). The effect of plasma-activated water combined with rosemary extract (*Rosmarinus officinalis* L.) on the physicochemical properties of Frankfurter sausage during storage. Foods.

[14] Wu M., Ma Y., Dou X., Aslam M.Z., Liu Y., Xia X., Yang S., Wang X., Qin X., Hirata T. (2023). A review of potential antibacterial activities of nisin against Listeria monocytogenes: The combined use of nisin shows more advantages than single use. Food Res. Int..

[15] Gao S., Zhai X., Wang W., Zhang R., Hou H., Lim L.-T. (2022). Material properties and antimicrobial activities of starch/PBAT composite films incorporated with ε-polylysine hydrochloride prepared by extrusion blowing. Food Packag. Shelf Life.

[16] Dixit A., Sabnis A., Balgude D., Kale S., Gada A., Kudu B., Mehta K., Kasar S., Handa D., Mehta R. (2023). Synthesis and characterization of citric acid and itaconic acid-based two-pack polyurethane antimicrobial coatings. Polym. Bull..

[17] Kumaresan V., Bhatt P., Arockiaraj J. (2016). Membrane disruption antimicrobial mechanism of Channa striatus lysozyme-derived antimicrobial peptides (AMP). Fish Shellfish Immunol..

[18] Nassar R., Nassar M., Vianna M.E., Naidoo N., Alqutami F., Kaklamanos E.G., Senok A., Williams D. (2021). Antimicrobial activity of phytic acid: An emerging agent in endodontics. Front. Cell. Infect. Microbiol..

[19] Che Lah N.A., Kamaruzaman A. (2024). The physico-chemical and antimicrobial properties of nano ZnO functionalised tannic acid. Sci. Rep..

[20] Du Y., Mi S., Wang H., Yang F., Yu H., Xie Y., Guo Y., Cheng Y., Yao W. (2023). Inactivation mechanism of Alternaria alternata by dielectric barrier discharge plasma and its quality control on fresh wolfberries. Food Control.

[21] Zhang J., Hu Z., Chen D., Yu Z., Huang L., Yu H., Yao W., Xie Y. (2023). Inactivation effect of Staphylococcus aureus and application on fresh-cut pineapples by plasma-activated tartaric acid. Food Biosci..

[22] Kong J., Zhang Y., Ju J., Xie Y., Guo Y., Cheng Y., Qian H., Quek S.Y., Yao W. (2019). Antifungal effects of thymol and salicylic acid on cell membrane and mitochondria of Rhizopus stolonifer and their application in postharvest preservation of tomatoes. Food Chem..

[23] Dong H., Han S., Mi K., Hao Y., Waterhouse G.I., Tong L., Hou S. (2025). Asymmetric Janus composite films with superior humidity regulation capabilities for the efficient preservation of strawberry fruit. Food Chem..

[24] Yang X., Zhang C., Li Q., Cheng J.-H. (2023). Physicochemical properties of plasma-activated water and its control effects on the quality of strawberries. Molecules.

[25] Zhang D., Liu Y., Li X., Xiao J., Sun J., Guo L. (2022). Inactivation of Escherichia coli on broccoli sprouts via plasma activated water and its effects on quality attributes. LWT-Food Sci. Technol..

[26] Dong G., Liu H., Yu X., Zhang X., Lu H., Zhou T., Cao J. (2018). Antimicrobial and anti-biofilm activity of tannic acid against Staphylococcus aureus. Nat. Prod. Res..

[27] Slabbert N.E., Hemingway R.W., Laks P.E. (1992). Complexation of Condensed Tannins with Metal Ions. Plant Polyphenols.

[28] Yao Q., Xu H., Zhuang J., Cui D., Ma R., Jiao Z. (2023). Inhibition of fungal growth and aflatoxin B1 synthesis in aspergillus flavus by plasma-activated water. Foods.

[29] Wang J., Sheng Z., Liu Y., Chen X., Wang S., Yang H. (2023). Combined proteomic and transcriptomic analysis of the antimicrobial mechanism of tannic acid against Staphylococcus aureus. Front. Pharmacol..

[30] Tintino S.R., Oliveira-Tintino C.D., Campina F.F., Silva R.L., Costa M.D.S., Menezes I.R., Calixto-Júnior J.T., Siqueira-Junior J.P., Coutinho H.D., Leal-Balbino T.C. (2016). Evaluation of the tannic acid inhibitory effect against the NorA efflux pump of Staphylococcus aureus. Microb. Pathog..

[31] Borisov V.B., Siletsky S.A., Nastasi M.R., Forte E.R.O.S. (2021). defense systems and terminal oxidases in bacteria. Antioxidants.

[32] Li B., Song Z., Zhang M., Ma Q., Hu W., Ding C., Chen H. (2025). Study on the damage and variation of Agropyron mongolicum induced by the combined action of discharge plasma and plasma-activated water. Plant Physiol. Biochem..

[33] Duan B., He Y., Hao H., Wang L., Zhang L., Wang Y., Liu C., Li Y., Lu K., Yin X. (2025). Preparation of curdlan/tannin acid composite hydrogels with photothermal conversion, antibacterial and antioxidant properties. Colloids Surf. A Physicochem. Eng. Asp..

[34] Jyung S., Kang J.-W., Kang D.-H. (2023). Inactivation of Listeria monocytogenes through the synergistic interaction between plasma-activated water and organic acid. Food Res. Int..

[35] Saidji N., Malki F., Boukerche H., Mokrane H. (2024). Insight into stability and degradation kinetics of Roselle (*Hibiscus sabdariffa* L.) flowers anthocyanin, effect of pH, heating, storage conditions, and co-pigment treatment. Biomass Convers. Biorefinery.

[36] Song C., Wang J., Wu L., Liu J., Liu G., Gong D., Zhang W., Wei J., Zhang Z. (2025). Quality and physiological changes in fresh-cut mango fruit as affected by cold plasma-activated water. Postharvest Biol. Technol..

[37] Zhao L., Li H., Wang K., Li X., Guo C., Yang H. (2022). Effects of electrolysed water and levulinic acid combination on microbial safety and polysaccharide nanostructure of organic strawberry. Food Chem..

[38] Ma R., Yu S., Tian Y., Wang K., Sun C., Li X., Zhang J., Chen K., Fang J. (2016). Effect of non-thermal plasma-activated water on fruit decay and quality in postharvest Chinese bayberries. Food. Bioprocess Technol..

[39] Perinban S., Orsat V., Raghavan V. (2022). Influence of plasma activated water treatment on enzyme activity and quality of fresh-cut apples. Food Chem..

